# A High-Speed Target-Free Vision-Based Sensor for Bus Rapid Transit Viaduct Vibration Measurements Using CMT and ORB Algorithms

**DOI:** 10.3390/s17061305

**Published:** 2017-06-06

**Authors:** Qijun Hu, Songsheng He, Shilong Wang, Yugang Liu, Zutao Zhang, Leping He, Fubin Wang, Qijie Cai, Rendan Shi, Yuan Yang

**Affiliations:** 1 School of Civil Engineering and Architecture, Southwest Petroleum University, Chengdu 610500, China; 201231010027@swpu.edu.cn (Q.H.); 201521000732@stu.swpu.edu.cn (S.H.); 201231010028@swpu.edu.cn (L.H.); 201521000730@stu.swpu.edu.cn (R.S.); 201521000729@stu.swpu.edu.cn (Y.Y.); 2 School of Transportation and Logistics, Southwest Jiaotong University, Chengdu 610031, China; wangshilong@my.swjtu.edu.cn (S.W.); caiqijieswjt@my.swjtu.edu.cn (Q.C.); 3 School of Mechanical Engineering, Southwest Jiaotong University, Chengdu 610031, China; zzt@swjtu.edu.cn; 4 School of Information Science and Technology, Southwest Jiaotong University, Chengdu 610031, China; fbwwang@my.swjtu.edu.cn

**Keywords:** vision-based sensor, vibration measurement, structural health monitoring, keypoint matching, CMT, BRT viaducts

## Abstract

Bus Rapid Transit (BRT) has become an increasing source of concern for public transportation of modern cities. Traditional contact sensing techniques during the process of health monitoring of BRT viaducts cannot overcome the deficiency that the normal free-flow of traffic would be blocked. Advances in computer vision technology provide a new line of thought for solving this problem. In this study, a high-speed target-free vision-based sensor is proposed to measure the vibration of structures without interrupting traffic. An improved keypoints matching algorithm based on consensus-based matching and tracking (CMT) object tracking algorithm is adopted and further developed together with oriented brief (ORB) keypoints detection algorithm for practicable and effective tracking of objects. Moreover, by synthesizing the existing scaling factor calculation methods, more rational approaches to reducing errors are implemented. The performance of the vision-based sensor is evaluated through a series of laboratory tests. Experimental tests with different target types, frequencies, amplitudes and motion patterns are conducted. The performance of the method is satisfactory, which indicates that the vision sensor can extract accurate structure vibration signals by tracking either artificial or natural targets. Field tests further demonstrate that the vision sensor is both practicable and reliable.

## 1. Introduction

Civil engineering structures are the main bodies that resist loads. During their operational life, civil engineering structures are exposed to various external loads, such as traffic, wind gusts, and seismic loads. These external loads are the main reason for the degradation of the structures. Health monitoring on major civil engineering structures has become an important research topic. At present, structural health monitoring (SHM) is carried out through the installation of contact sensors and their corresponding data acquisition systems. Such an approach, however, has many limitations such as installation difficulty, and being time-consuming, and high cost. Specifically, installation of these contact sensors often interrupts the normal operation of the structure. Therefore, it is necessary to develop a more effective and practical SHM method. The first bus rapid transit (BRT) line was built in Curitiba (Brazil) in 1974. After that, the new transportation method spread rapidly all over the world and has now become an indispensable part of urban traffic. As one of the main modes of transportation in big cities, such as Chengdu (China), the BRT viaduct use cannot be interrupted in view of the heavy traffic and security. In this case, traditional sensing systems are not easily implemented.

At present, structural vibration response can be applied to the operational state analysis [1,2,3] of existing bridges. Furthermore, damage identification [4,5,6] and life prediction calculations [7,8] can also be carried out. Such a method has become an active research field owing to its excellent performance and the fact that it requires very few parameters. Therefore, it is of great significance to measure the vibrations precisely, rapidly, and economically. Currently available sensors for measuring structural vibrations can be classified into contact and non-contact sensors. Contact sensors, such as accelerometers [9,10], linear variable differential transformers (LVDT) [11], and strain-type displacement sensors (STDS) [12] are widely used in monitoring systems to obtain valuable structure vibration information. Non-contact sensors such as global position system (GPS) [13], laser Doppler vibrometers [14], and radar interferometry system [15] are less used because they are expensive, complex, and not very accurate [16,17,18].

Vision-based vibration measurement systems are burgeoning. They are gradually replacing conventional vibration measurement sensors owing to their relatively low cost as well as flexible and convenient installation, especially for target-free vision-based sensor approaches. Various techniques have been implemented for moving object tracking and displacement measurement, such as template matching [19,20], optical flow field [21,22], frame differential method [23], and digital image correlation method (DICM) [24,25]. The optical flow is greatly affected by different light intensities, making it not very applicable to the field. The frame differential method is only used to determine whether an object is moving in an area or not and cannot extract the full image of the moving objects. The digital image cross-correlation is a measurement method for the analysis of the entire field displacement and strain, however, it cannot measure local vibrations.

The most frequently used method is template matching, which can be categorized into three types based on its template styles, namely, global template matching, local template matching and keypoint matching. The first two matching methods have good precision, however their efficiency is low because of their high consumption of time and random-access memory (RAM). The keypoints matching method can overcome this deficiency, and thus, this method has been widely studied. A variety of keypoints have been detected and descriptor algorithms have been proposed, such as scale-invariant feature transform (SIFT) [26], speeded-up robust features (SURF) [27], features from accelerated segment test (FAST) [28,29], adaptive and generic accelerated segment test (AGAST) [30], binary robust invariant scalable keypoints (BRISK) [31] and ORB [32]. Among these algorithms, the ORB algorithm is very popular for the reason that it has the best efficiency and rotational invariance and its scale invariance is retained. It consists of two components—oFAST and rBRIEF—which have improved performance compared to the FAST keypoint detector and Binary Robust Independent Elementary Features(BRIEF) [33] descriptors. The ORB algorithm is nearly two orders of magnitude faster than the SIFT one [34], and one order faster than the SURF one [33]. Thus, a number of object tracking algorithms have been proposed, such as tracking learning detection (TLD) [35], visual tracking decomposition (VTD) [36], incremental visual tracking (IVT) [37], multi-task tracking (MTT) [38], visual tracker sampler (VTS) [39], and CMT [40,41]. The CMT algorithm was proposed by Nebehay et al. in 2014. It employs a novel consensus-based scheme for outlier detection in the voting behavior to eliminate erroneous keypoints. In this method, the number of keypoints has been reduced, while the process becomes more efficient.

Although computer vision measurement technology is still in its infancy, some achievements have been recorded and it has great prospects for the future. In this study, we propose a novel vision-based sensor for BRT viaduct vibration measurement employing CMT combined with ORB algorithm. In practical application, the primary concern for vision-based sensor is the measurement efficiency which mainly refers to the accuracy and operating speed. To improve the accuracy of object orientation, keypoint matching technology was employed to seek the latent object point, meanwhile voting and consensus were applied for removing the outliers. A more efficient combination pair of detector and descriptor was further tested to improve the execution speed of the algorithm based on the aforementioned technology. In general, the proposed vision sensor has the following properties: (1) easy to install and set up, without pre-installed artificial targets; (2) the measurement efficiency of algorithm is higher than that of existing algorithms, which means that the sensor is well adapted to high-speed monitoring systems; (3) precision is kept at a good level.

This study aims at solving vibration sensing and measuring problems through the vision sensor method. To address these challenges, three key steps were employed, namely, preprocessing, object tracking, and vibration analysis. Homomorphic filtering was introduced for preprocessing, tracking of objects was realized using an improved CMT object recognition and tracking algorithm, resulting in an improved method for calculation of scaling factors and a more accurate vibration analysis. A series of laboratory tests were conducted to evaluate the reliability of this method. Furthermore, the vibration measurement of a BRT viaduct in Chengdu (China) was selected as a case study to illustrate the specific process of the vision sensor method. Finally, field test results were used to validate this method.

## 2. Proposed Vision Sensing Approach

**Figure 1:**
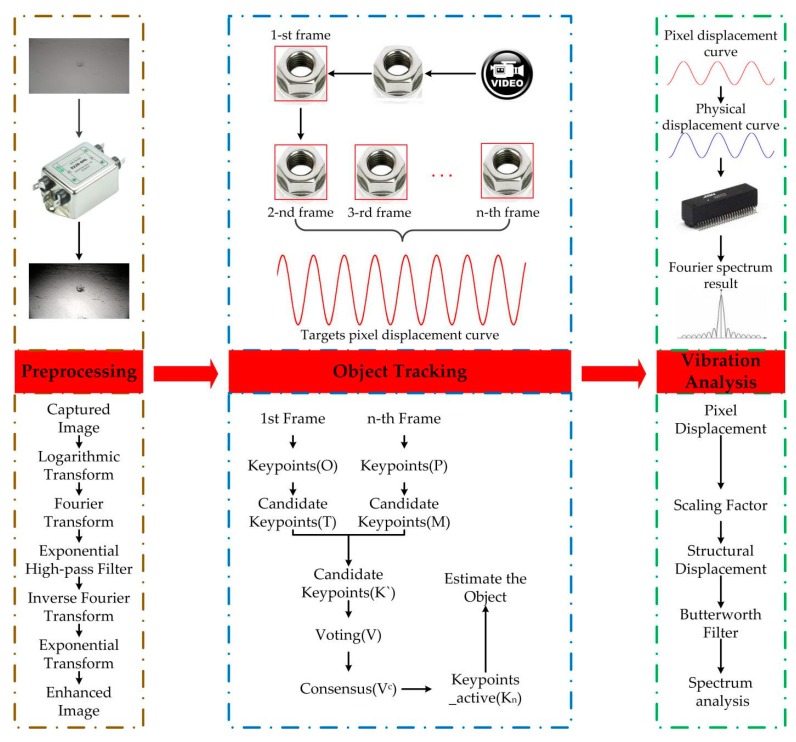
Vision sensor implementation procedure.

In this study, the basic principle of the vision-based sensor for vibration displacement measurement is the keypoints matching technology. The proposed methodology mainly includes three steps: preprocessing of captured video, free-target tracking using the combination of CMT tracking algorithm and ORB keypoints detector, and vibration analysis, as illustrated in Figure 1.

### 2.1. Preprocessing of Captured Video

In order to improve the identifiability of an object selected arbitrarily in this study, pretreatment of the captured video was performed at the beginning because the vision-based sensor was considered well suited for harsh field environments that are not properly illuminated. Brightness and contrast adjustment are simple and effective tasks that can be employed for preprocessing. Homomorphic filtering [42] has been incorporated into contrast enhancement and consists of the following steps:

Step One: The basic nature of the image *F*(*x, y*) can be naturally described as:
  (1) F(x,y)=i(x,y)r(x,y),
  where *i*(*x*, *y*) and *r*(*x*, *y*) denote the illumination and reflection components respectively, 0 < *i*(*x*, *y*) < ∞ and 0 < *i*(*x*, *y*) < 1.
Step Two: Because the Fourier transform of the product of two functions is not separable, logarithmic transformation is employed to solve the problem. Define:
  (2) z(x,y)=lnF(x,y)=lni(x,y)+lnr(x,y).
  Then:
  (3) F(z(x,y))=F(lnF(x,y))=F(lni(x,y))+F(lnr(x,y)),
  where *F*() denotes the Fourier transform.
  Equation (3) can be written as:
  (4) Z(u,v)=I(u,v)+R(u,v),
  where *Z*(), *I*(), *R*() are the Fourier transforms of *z*(), *lni*(), and *lnr*() respectively.
Step Three: A homomorphic filter is applied to suppress low frequency components and enhance high frequency components. Thus:
  (5) S(u,v)=H(u,v)Z(u,v)=H(u,v)I(u,v)+H(u,v)R(u,v),
  we apply a exponential high-pass filter (EHPF) to the method as follows:
  (6) $$D(u,v)=\sqrt{{\left(u-x_{0}\right)}^{2}+{\left(v-y_{0}\right)}^{2}},$$
  (7) $$H(u,v)=(H_{h}-H_{l})\times (1-e^{-c{\left(\frac{D(u,v)}{D_{0}}\right)}^{4}})+H_{l},$$
  where 1< *u* < *M*, 1 < *v* < *N*, *M* and *N* denote the number of pixels on the x- and y-axes. *x*_0_ = *floor*(*M/2*), *y*_0_ = *floor*(*N/2*), and *floor*() are rounded down. *H_h_*, *H_l_*, *c*, *D*_0_ are the filter parameters that must be entered by users. All of these parameters are selected by a human visual system (HVS). After several tests, the values were identified as *H_h_* = 1.0, *H_l_* = 1.5, *c* = 1.5, *D*_0_ = 1.0.
Step Four: Using an inverse Fourier transform, the processed image is reconstructed. This can be obtained using:
  (8) $$s(x,y)=F^{-1}(S(u,v))=F^{-1}(H(u,v)I(u,v))+F^{-1}(H(u,v)R(u,v)),$$
  by defining:
  (9) $$i^{\prime }(x,y)=F^{-1}(H(u,v)I(u,v)),$$
  and:
  (10) $$r^{\prime }(x,y)=F^{-1}(H(u,v)R(u,v)),$$
  we get:
  (11) $$s(x,y)=i^{\prime }(x,y)+r^{\prime }(x,y),$$
  where *F^−^*^1^() denotes the inverse Fourier transform.
Step Five: The inverse yields the desired enhanced image *g*(*x*, *y*), that is:
  (12) $$g(x,y)=\exp(s(x,y))=g_{i}(x,y)g_{r}(x,y),$$
  where *g_i_*(*x*, *y*) and *g_r_*(*x*, *y*) denote the enhanced illumination and reflection components respectively.

**Figure 2:**
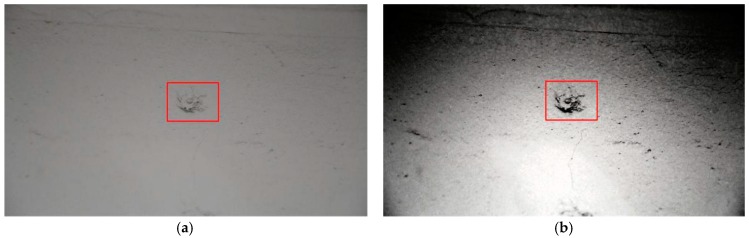
Effects of preprocessing: (**a**) Original image; (**b**) Preprocessed image.

As shown in Figure 2, the enhancing algorithm can significantly improve the quality of the images as the object becomes easier to capture.

### 2.2. Improved CMT Algorithm for Object Tracking

In this study, the proposed vision sensor was developed based on CMT, a keypoint-based method for object tracking first described Nebehay et al. Image sequences *I*_1_, *I*_2_,…,*I_n_* and an initializing region *b*_1_ in *I*_1_ are the input of this object tracking system, and the system returns subsequent region *b*_2_…,*b_n_* in *I*_2_,…,*I_n_*. In this process, voting and consensus are the core of the algorithm as described in the following procedure.

Step One: The ORB detector is employed to detect the keypoints located in the initialization region and described using the BRISK descriptor to initialize a set of keypoints *O*, followed by a mean normalization of the keypoints locations. In each frame, the set of keypoints *O* is used for matching; this will assist in recognizing the object when it re-enters the visual field.
Step Two: In each frame *t*, we are interested in finding a set of corresponding keypoints *K_t_* to represent the object as accurately as possible. Two complementary methods are presented for investigation: optical flow and keypoint-based method. Similarly, a set of candidate keypoints *P* can be established using the ORB algorithm. On the other hand, another set of candidate keypoints *T* is obtained by the option flow method, As a result *K_t−_*_1_ contains only the keypoints located in region box in the previous frame; therefore, the set of candidate keypoints *T* do not include the keypoints exist in background.
Step Three: The set of keypoints *M* is obtained by matching the candidate keypoints *P* with the keypoints *O*. By doing this, the background keypoints are removed from *M*.
Step Tour: The next step is to fuse *T* and *M* into a set of keypoints *K'*. Tracked keypoints are removed when there exists a match associated with the same model keypoint, this results in a more robust matched keypoints. It is worth mentioning that the outliers still exist.
Step Five: In the CMT algorithm, voting is implemented to relocate the object in each frame. Each keypoint in *K'* casts a vote for the object center, resulting in a set of votes:
  (13) $$V={\left\{h(a_{i},m_{i})\right\}}_{i=1}^{N^{K^{\prime }}},$$
  where *a_i_* refers to the keypoint position in image coordinates and *m_i_* = ($x_{i}^{0}$, $x_{i}^{t}$) are the index of the corresponding keypoints in frame *t*, in which $x_{i}^{t}$ denotes the position of $x_{i}^{0}$ in frame *t*. We consider translational, scale, and rotational changes of the object:
  (14) $$h(a,m)=a-s\cdot Rr_{m},$$
  where *r_m_* is the relative position of the corresponding keypoints in *O*, *s* is the scale factor given by:
  (15) $$s=med\left(\left\{\frac{\left\|x_{i}^{t}-x_{j}^{t}\right\|}{\left\|x_{i}^{0}-x_{j}^{0}\right\|},i\neq j\right\}\right),$$
  where *med* is the median, and *R* is a *2D* rotation matrix given by:
  (16) $$R=\left(\begin{array}{cc} \cos\alpha & -\sin\alpha \\ \sin\alpha & \cos\alpha \end{array}\right).$$

The rotation α can be obtained using the function *a*tan 2 as described in reference [41]:

(17) $$\alpha =med\left(\left\{a\tan2(x_{i}^{0}-x_{j}^{0})-a\tan2(x_{i}^{t}-x_{j}^{t}),i\neq j\right\}\right).$$

Step Six: The outlier keypoints can be removed by consensus. Just as in the CMT, the hierarchical agglomerative clustering based on the Euclidean distance is applied to cluster the correspondences. The consensus cluster *V^c^* is identified according to the highest number of votes, and the active keypoints *K_t_* is the subset of *K'* that voted into *V^c^*; it is used for the next cycle to acquire the *K_t+_*_1_.
Step Seven: The bounding boxes can be derived by:
  (18) $$b_{t}=f(b_{1},\mu_{t},s_{t},\alpha_{t}),$$
  where *b***_1_** is the initializing region, μ***_t_*** is the object center in ***t*** frame and it can be obtained by:
  (19) $$\mu =\frac{1}{n}\sum_{i=1}^{n}V_{i}^{c},$$
  where *n* is the element number of *V^c^*, *s_t_* is the scale factor in *t* frame, and α***_t_*** is the rotation factor in t frame.

**Figure 3:**
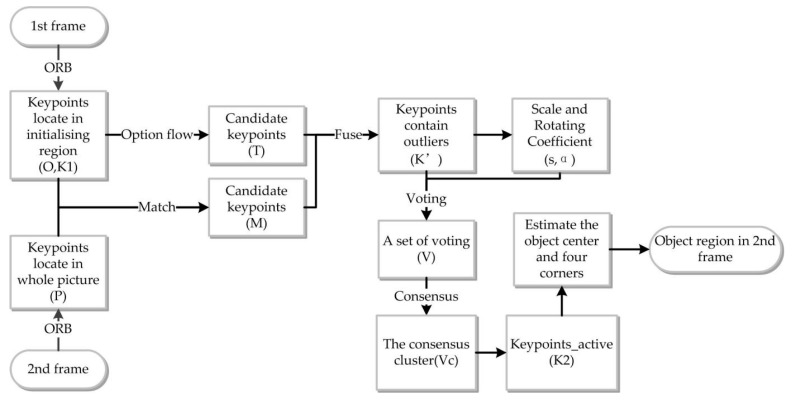
Procedure for object region identification in the second frame.

To describe the process better, the procedure for object region (*b*_2_) identification in the second frame is shown in Figure 3. In this algorithm, the keypoints detector and descriptor have a significant impact on the operational efficiency of the procedures. In this section, we report the results of a comparative analysis of the different keypoint detector and descriptor algorithms pairs. For the FAST, ORB, BRISK and AGAST detectors and the ORB and BRISK descriptors, the processing time, the computational cost, and the matching accuracy were evaluated in order to compare the performance of the different algorithms.

#### 2.2.1. Processing Time

**Figure 4:**
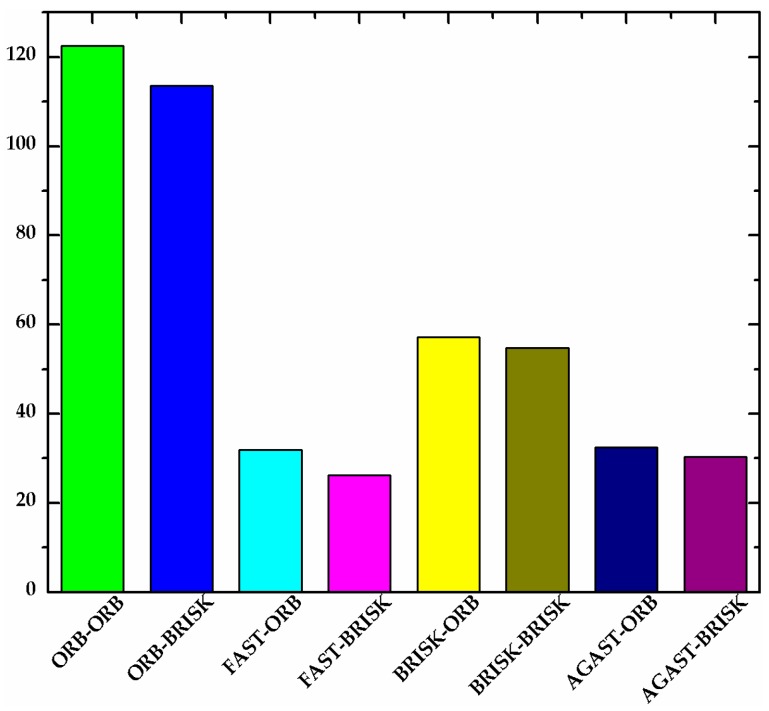
Average number of active keypoints for a sample video with different combinations of detectors and descriptors.

The detectors and descriptors were evaluated using the processing time metric. As a general rule, the processing time depends on the number of detected keypoints and the complexity of the input image. For the evaluation, a video with an artificial target was used. Figure 4 shows the average number of active keypoints for a sample video with different combinations of detectors and descriptors. As shown in Figure 4, the average number of active keypoints detected by ORB and BRISK were higher than those detected by FAST and AGAST. In general, the number of active keypoints has an impact on tracking stability. On the other hand, the processing time of single keypoint tracking is listed in Table 1. The result shows that the best validity and efficiency were achieved by using the ORB detector algorithm. As can be seen from Figure 4, the number of keypoints which are detected by ORB is far larger than that detected by other detectors, which means that using ORB detector ensures stability of target tracking. On the other hand, Table 1 shows that the durations for tracking a keypoint was 3.8349 and 2.7244 ms, respectively. Both are minimum processing time of the different combination pairs, which proves the efficiency of this algorithm. 

#### 2.2.2. Computational Cost

To evaluate the computational cost, different combination pairs were tested. The processor time, defined as the percentage of processor execution time for idle threads, was evaluated in order to determine the central processing unit (CPU) utilization. Table 2 presents the average processor time for the sample video. It was observed that the ORB detector was more efficient compared with BRISK, FAST, and AGAST. For instance, the average processor time of ORB is less than those of BRISK, AGAST, and FAST by a factor of 1, 2, and 2.5, respectively.

#### 2.2.3. Matching Accuracy

The matching accuracy criterion was introduced, similar to the one proposed in [43], and is defined as the ratio between the number of correct matches and the total number detected:

(20) $$accuracy=\frac{n}{N}\times 100\%,$$

where *n* denotes the number of correct matches and *N* is the total number of matches. The number of false matches relative to the total number detected is given by:

(21) $$1-accuracy=\frac{N-n}{N}\times 100\%,$$

therefore, the 1-*accuracy* can be calculated. Table 3 lists the 1-*accuracy* values. The table indicates that the BRISK detector had the best results; the BRISK descriptor yielded the best results irrespective of the implemented detector, while the ORB detector yielded the second best results.

To evaluate the performance of different combination pairs in order to obtain scientific and constant experimental data, a dimensionless parameter was introduced as:

(22) $$X^{*}=\frac{X-\min}{\max-\min},$$

where *X*^∗^ denotes the normalized values of the parameter, *X* denotes the sample data, max and min denote the maximum value and minimum value of the sample data, respectively.

**Figure 5:**
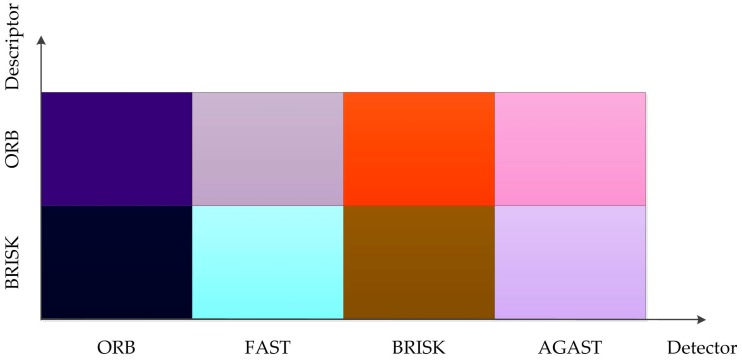
Performance comparison of different combination pairs.

In order to illustrate the impact of all these parameters on the computing performance, the comprehensive performances of different combination pairs were represented with colors. The dimensionless result of *processing time*, *processor time*, and 1-*accuracy* were represented with color values in R, G and B channels, respectively. As shown in Figure 5, colors were used to represent the computing performance of different combination pairs. For all the parameters, the lower the value, the better the computing performance, and the darker the color, the better the performance. It is clear that our choice of the combination of ORB detector and BRISK descriptor gave the best performance.

### 2.3. Scaling Factor Determination

**Figure 6:**
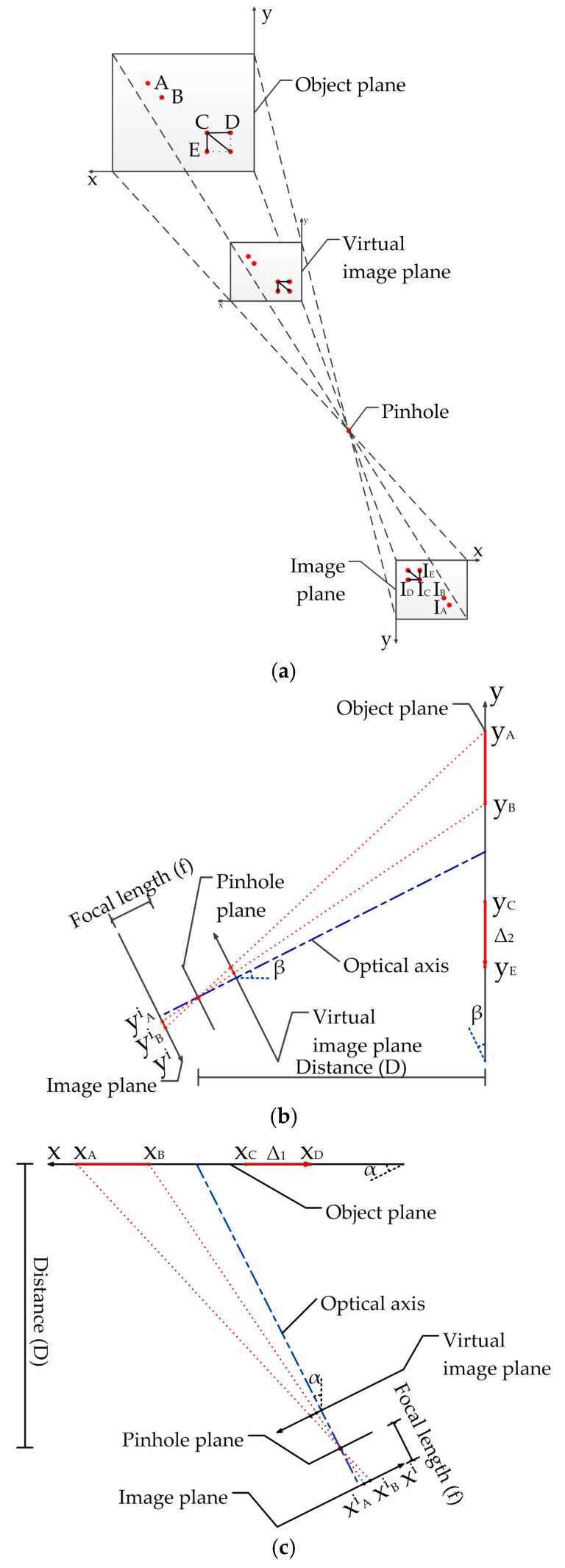
Error analysis of scaling factor: (**a**) 3D view of image plane and object plane; (**b**) Optical axis non-perpendicular to object plane in the vertical direction; (**c**) Horizontal direction.

From the captured video, the pixel length at the image plane can be obtained. In order to obtain the structural displacements, the relationship between the pixel coordinate and the physical coordinate should be established. According to the method developed by Feng et al. [44], two calculation methods for scaling factor have been made a detailed introduction in [44]. Therefore, the following equations can be deduced based on the above calculation processes according to [44]:

(23) $$SF_{1}=\frac{y_{A}-y_{B}}{I_{A}^{iy}-I_{B}^{iy}},$$

(24) $$SF_{2}=\frac{D}{f\cos^{2}\theta }\cdot d_{pixel},$$

where *y_A_* and *y_B_* are the coordinates of the two points on the object surface as shown in Figure 6; $I_{A}^{iy}$ and $I_{B}^{iy}$ are the corresponding pixel coordinates at the image plane, which can be computed using the captured video, *D* is the distance between the camera and the object along the optical axis, *f* is the focal length, *θ* is the angle between the camera optical axis and the normal directions of the object surface, namely, *α* and *β*; *d_pixel_* is the pixel size (e.g., in *mm/pixel*). *y_A_* and *y_B_* can be calculated as follows:

(25) $$y_{A}=\frac{Dy_{A}^{i}}{f\;\cos^{2}\theta -y_{A}^{i}\cos\theta \sin\theta },$$

(26) $$y_{B}=\frac{Dy_{B}^{i}}{f\;\cos^{2}\theta -y_{B}^{i}\cos\theta \sin\theta },$$

where $y_{A}^{i}=I_{A}^{iy}$·*d_pixel_* and $y_{B}^{i}=I_{B}^{iy}$·*d_pixel_* are the coordinates at the image plane.

In addition, the error calculation formula is given in [44]. For example, if the object point has a small displacement Δ in Figure 6, it can be decomposed into Δ_1_ along the *x*-axis and Δ_2_ along the *y*-axis at the object surface. Similarly, *x_C_* and *x_D_* are the coordinates of the two points in the *x*-axis, and *y_C_* and *y_E_* are the coordinates of the two points in the *y*-axis. These coordinates can be calculated by Equations (25) and (26). The “true displacement” Δ_1_ and Δ_2_ are considered to be the distances between corresponding points, which are that:

(27) $$\Delta_{1}=x_{C}-x_{D}=\frac{Dx_{C}^{i}}{f\;\cos^{2}\alpha -x_{C}^{i}\cos\alpha \sin\alpha }-\frac{Dx_{D}^{i}}{f\;\cos^{2}\alpha -x_{D}^{i}\cos\alpha \sin\alpha },$$

(28) $$\Delta_{2}=y_{C}-y_{E}=\frac{Dy_{C}^{i}}{f\;\cos^{2}\beta -y_{C}^{i}\cos\beta \sin\beta }-\frac{Dy_{E}^{i}}{f\;\cos^{2}\beta -y_{E}^{i}\cos\beta \sin\beta },$$

where $x_{C}^{i}=I_{C}^{ix}$·*d_pixel_*, $x_{D}^{i}=I_{D}^{ix}$·*d_pixel_*, $y_{C}^{i}=I_{C}^{iy}$·*d_pixel_*, $y_{E}^{i}=I_{E}^{iy}$·*d_pixel_* are the coordinates of point *C* before and after translation at the image plane. From the scaling factors *SF*_1_ in Equation (23) and *SF*_2_ in Equation (24), the “measurement displacement” can be calculated as follows:

(29) $${\tilde{\Delta }}_{1}=(I_{C}^{i}-I_{D}^{i})\cdot SF_{1},$$

(30) $${\tilde{\Delta }}_{2}=(I_{C}^{i}-I_{D}^{i})\cdot SF_{2}.$$

Numerical methods were applied to quantify the error resulting from camera non-perpendicularity. The measurement error from the two scaling factors can be defined as:

(31) $$Error_{1}=\frac{\left({\tilde{\Delta }}_{1}-\Delta \right)}{\Delta }\times 100\%,$$

(32) $$Error_{2}=\frac{\left({\tilde{\Delta }}_{2}-\Delta \right)}{\Delta }\times 100\%$$.

**Figure 7:**
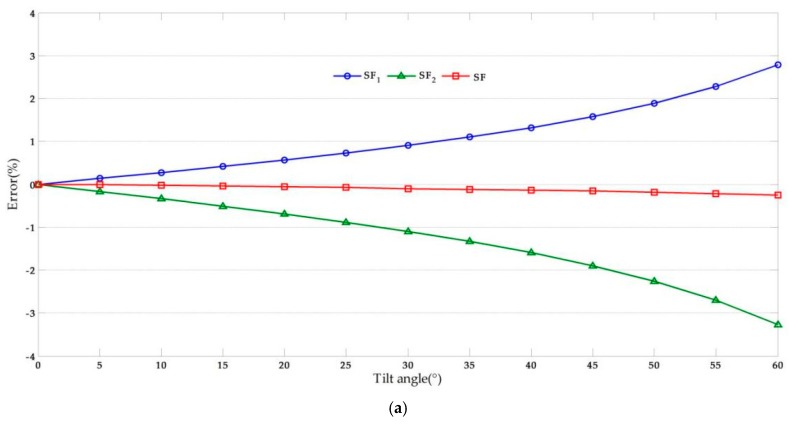

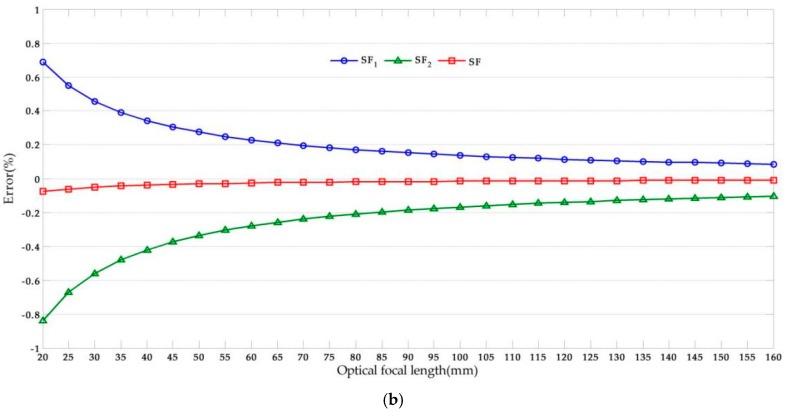
Error analysis results: (**a**) Effects of optical axis tilt angle (*f* = 50); (**b**) Effects of optical focal length (*θ* = 10°).

In this study, we used data from [44]. The following values were assigned: *d_pixel_ =* 4.8 µm, $I_{A}^{i}$ = 200, $I_{B}^{i}$ = 160, and *D* = 10 m. Point *C* has a 1 pixel translation both in the *x*-axis and *y*-axis at the image plane from $I_{C}^{i}$ = 100 to $I_{D}^{i}$ = 99. The effect of the tilt angle (*θ*) and lens focal length (*f*) were investigated by considering a variable range and the results are shown in Figure 7.

From Figure 7, we observe that the absolute value of the error increased with the increase of the tilt angle. Furthermore, the error varied inversely with the focal length and the effect was smaller than that of the tilt angle. Since that the error analysis method of the displacement in *x*-axis is similar to that of *y*-axis, then the error can be obtained by the same method.

In BRT structural vibration measurement, the vibration amplitude is only a few millimeters; therefore, the error should be minimized as much as possible. As expected, the calculation results of the scaling factor *SF*_1_ were larger than the “true displacement” while the calculation results of scaling factors *SF*_2_ were smaller. To reduce the error, we propose the use of a scaling factor *SF*, which can be obtained by:

(33) $$SF=\frac{1}{2}\times (\frac{x_{A}-x_{B}}{I_{A}^{i}-I_{B}^{i}}+\frac{D}{f\;\cos^{2}\theta }\cdot d_{pixel})$$

The error analysis results calculated using scaling factor *SF* are shown in Figure 7. It can be seen that the error due to camera non-perpendicularity decreased significantly while the validity improved. In the laboratory and field tests conducted in this study, the scaling factor *SF* was adopted. *x_A_* and *x_B_* can be obtained from field calibration and its corresponding image dimension in pixels $I_{A}^{i}$ and $I_{B}^{i}$, while the intrinsic parameters of the camera can be obtained from camera calibration [45].

**Figure 8:**
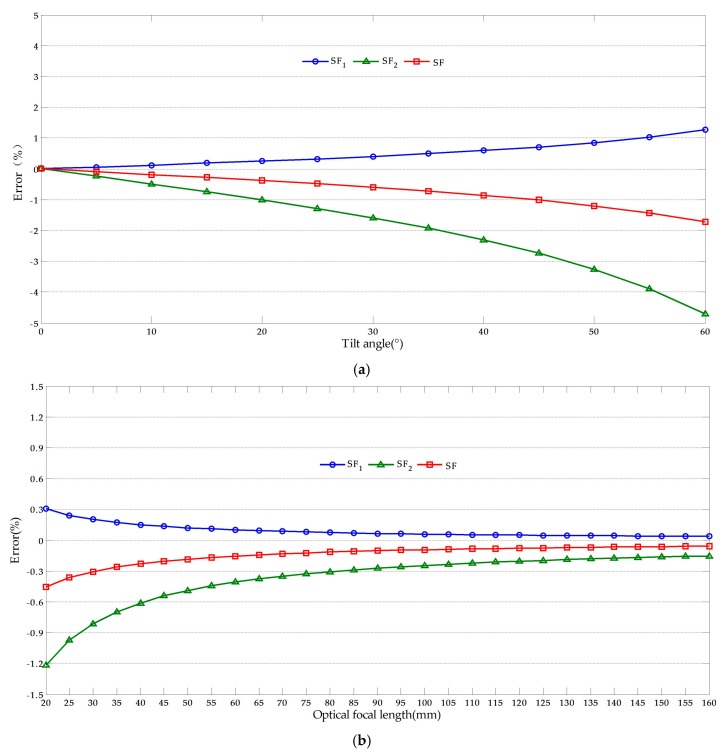
Error analysis results when the measurement point gets closer to the known dimension: (**a**) Effects of optical axis tilt angle (*f* = 50); (**b**) Effects of optical focal length (*θ* = 10°).

It is noteworthy that the measurement error from *SF*_1_ be decreased when the measurement point C gets closer to the known dimension AB, the results of the error analysis when *I_C_* = 190 to 189 as shown in Figure 8. This may lead to that the proposed methods fail to achieve the desired goal of reducing the measurement error. To solve this problem, the reference object could be kept farther away from the target objects, which enables the measurement point keep away from the known dimension. Normally, the target objects are located in the neighborhood of the captured video regional center while the reference object located in the upper-left or right corner of the video.

## 3. Hardware of the Vision Sensor System

As tabulated in Table 4, the proposed vision sensor system consists of three components: a video camera, optical lens, and laptop computer. The camera was fixed on a tripod during the test process. It was aimed at an arbitrary target, and captured the target within the shooting range of the video camera.

## 4. Laboratory Tests

### 4.1. Moving Platform Tests

The moving platform tests experiment was carried out to evaluate the performance of the vision-based sensor in a laboratory environment. The mechanical testing and simulation (MTS) electronic servo testsuite was used as the vibration source, and the motion was captured by the vision-based sensor and the strain-type displacement sensors (STDS). At present, STDS are widely used in displacement measurement of civil engineering structures. These apparatus work by the strain bridge principle. Specifically, the small deformation measured by the strain bridge and thus the mechanical quantity is changed into an electrical quantity. It has many advantages compared with traditional displacement sensors, for example, higher accuracy, wider range of measurement, longer service life, faster response speed, better frequency response, no environmental restrictions, cost-effectiveness and so on. Because it has an excellent performance in terms of small displacement measurements, the STDS is an optimum option in this experiment. In addition, the selection of vision sensor equipment should take into account vibration parameters and the working environment.

**Figure 9:**
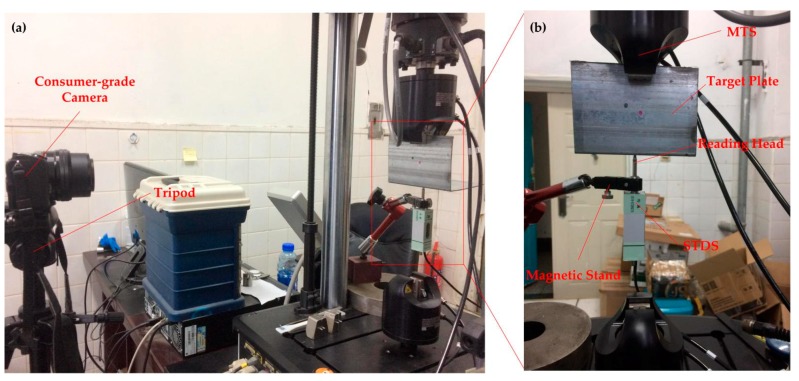
Setup for moving platform experiment: (**a**) Experimental setup; (**b**) Setup of target plant region.

Figure 9 shows the setup for the moving platform experiment. The target plate was installed on the CMT electronic servo TestSuite. The displacement sensor was installed on the target plate, with the magnetic stand fixed on it. The sensor of the measuring head maintained contact with the target plate. The camera head was installed on a tripod for steady output, and fixed at the right position to ensure that the target can be captured smoothly during the test duration.

**Figure 10:**
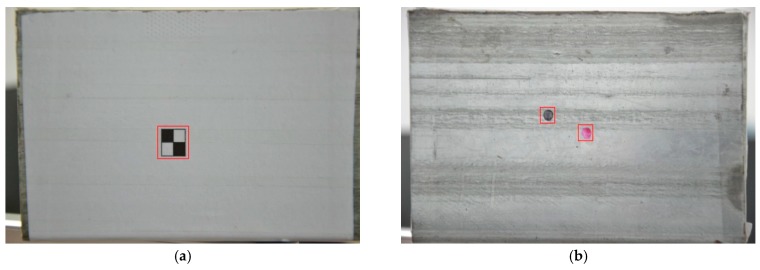
Target plate: (**a**) Artificial target; (**b**) Free targets.

Commissioning tests were carried out after equipment installation to determine the appropriate distance between the camera and target plate. The stability of the proposed algorithm is verified by designing two different types of target, as shown in Figure 10. Firstly, the artificial target is designed with significant characteristics, which is conductive to the achievement of continuous target tracking, but for the free targets, the colors, sizes and positions, are assigned randomly. In this way, the effectiveness and stability of the object tracking of arbitrary targets are confirmed. Secondly, the free target plant is designed with two different targets, which can be used to verify whether the error caused by human selection could have been prevented. Lastly, targets with different colors are employed to verify the color sensitivity of the algorithm.

Since that there are many vibration modes in the real environment, various frequencies, amplitudes and operating modes were applied to simulate the natural environment in a series of experiments. Table 5 and Table 6 list a series of low frequency vibration test parameter values. In addition, higher frequency vibration tests were designed to validate the performance; the parameter values are listed in Table 7.

In the laboratory experiment, the video camera was aimed at the target center, and made an angle *θ* = 0. The rest of the parameters are summarized in Table 8. Using the parameters and Equation (33), the scaling factor was obtained as *SF* = 0.138858.

To further evaluate the error performance and verify the precision and accuracy of the developed vision-based sensor, the normalized root mean squared error (NRMSE) was introduced as follows:

(34) $$NRMSE=\frac{\sqrt{\frac{1}{n}\sum_{n=1}^{n}{\left(x_{i}-y_{i}\right)}^{2}}}{y_{\max}-y_{\min}},$$

where *n* is the number of measurement data, *x_i_* and *y_i_* denote the *i*th displacement data at time *t_i_* measured by the vision sensor and the STDS, respectively, and *y_max_* = max(*y*), *y_min_* = min(*y*).

**Figure 11:**
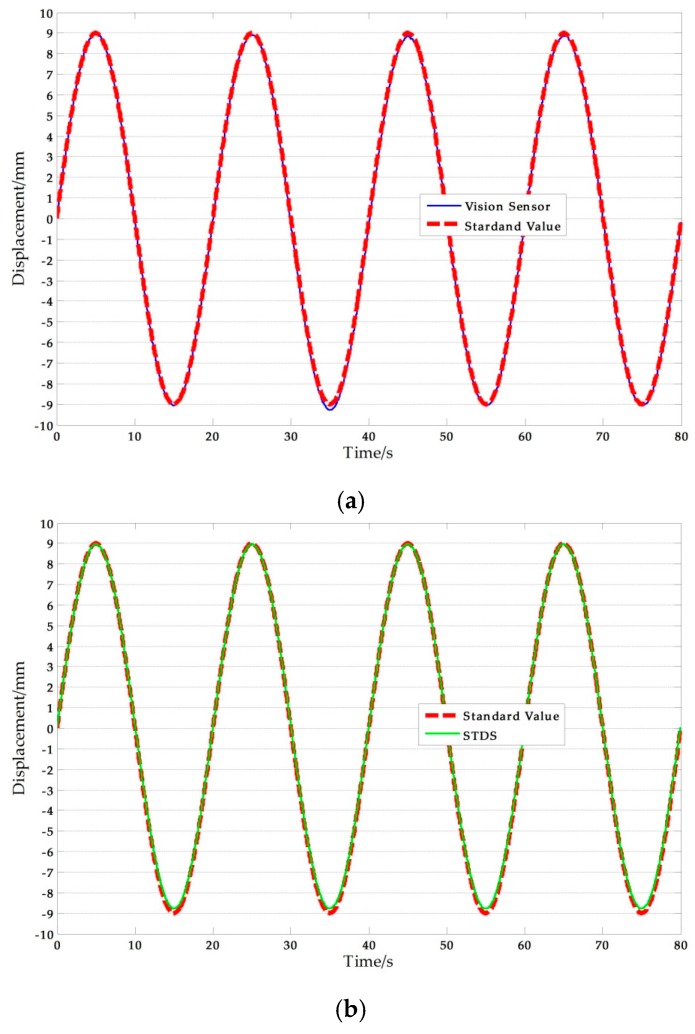
Artificial measurement comparisons: (**a**) Measurement results of vision-sensor compared with standard values; (**b**) Measurement results of STDS compared with standard values. (*f* = 0.05 Hz, *A* = 9 mm).

Figure 11 shows a set of experimental results obtained with the artificial target measurement test in Ι-5. The NRMSE errors were used in the analysis of the experimental data. The results are shown in Table 9, where the average NRMSE of the vision sensor measurement was 1.822%, and the maximum value was 3.041%. The average NRMSE of the displacement sensor measurement was 1.442%, and the maximum value was 3.433%.

**Figure 12:**
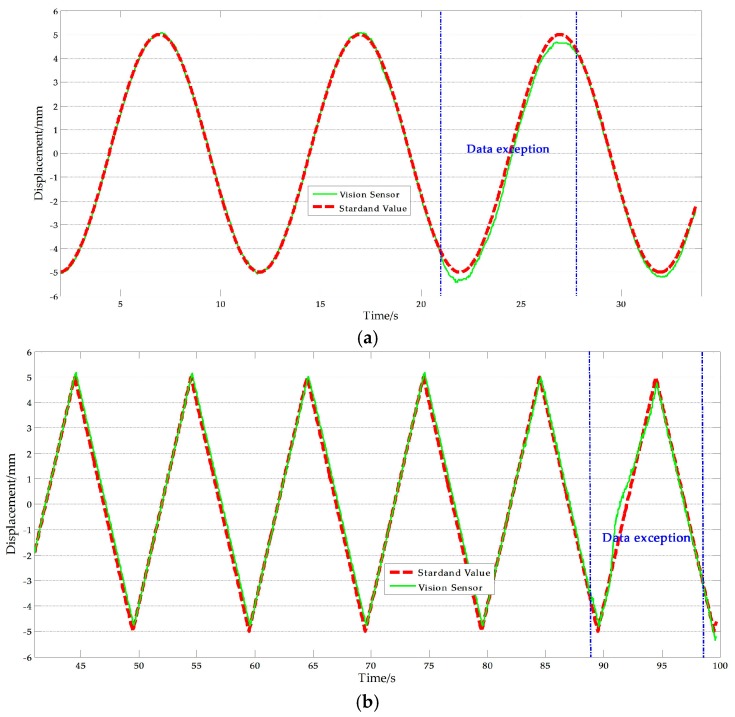
Exceptional artificial measurement results: (**a**) Ι-2; (**b**) Ι-4.

From Table 9, it can be noted that two sets of tests that have relatively big errors, 3.041% and 2.757% respectively. The reasons of this phenomenon can be obtained by analyzing the corresponding test data. Figure 12 shows the experimental results with artificial target measurement test of Ι-2 and Ι-4. As shown in the Figure 12, exceptional data with abnormal causes are present in some periods during the test duration which explains why the average NRMSE of the vision sensor measurement is larger than that of the displacement sensor measurement. The most likely cause of this anomaly is that unavoidable movement of the camera stand occurred. Removing the abnormal results, the average NRMSE of the vision sensor measurement was 1.315%, which implies that the improved vision-based sensor is consistent with traditional displacement sensor and therefore, suitable for actual measurements.

**Figure 13:**
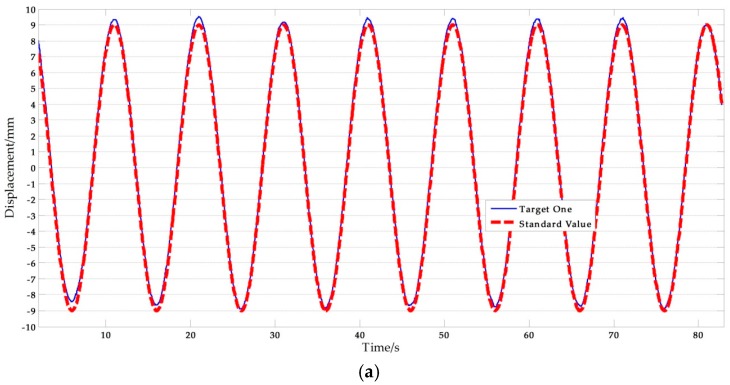

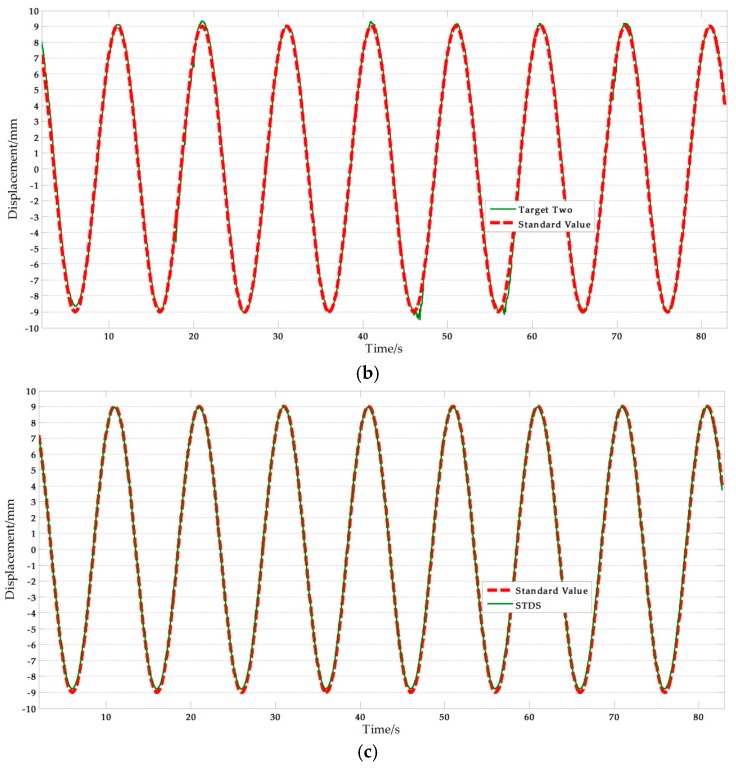
Free target measurement comparisons: (**a**) Measurement results of vision sensor capturing target one compared with standard values; (**b**) Measurement results of vision sensor capturing target two compared with standard values; (**c**) Measurement results of STDS compared with standard values. (*f* = 0.1 Hz, *A* = 9 mm).

Figure 13 shows a set of experimental results with free target measurement test in II-6. Table 10 presents the NRMSE errors analysis results. As presented in Table 10, the average NRMSE error of the vision-based sensor measurement was 1.805%, and the average NRMSE error of the displacement sensor measurement was 1.471%. Clearly, the vision-based sensor using a free target achieved a high accuracy comparable to traditional contact sensors. 

**Figure 14:**
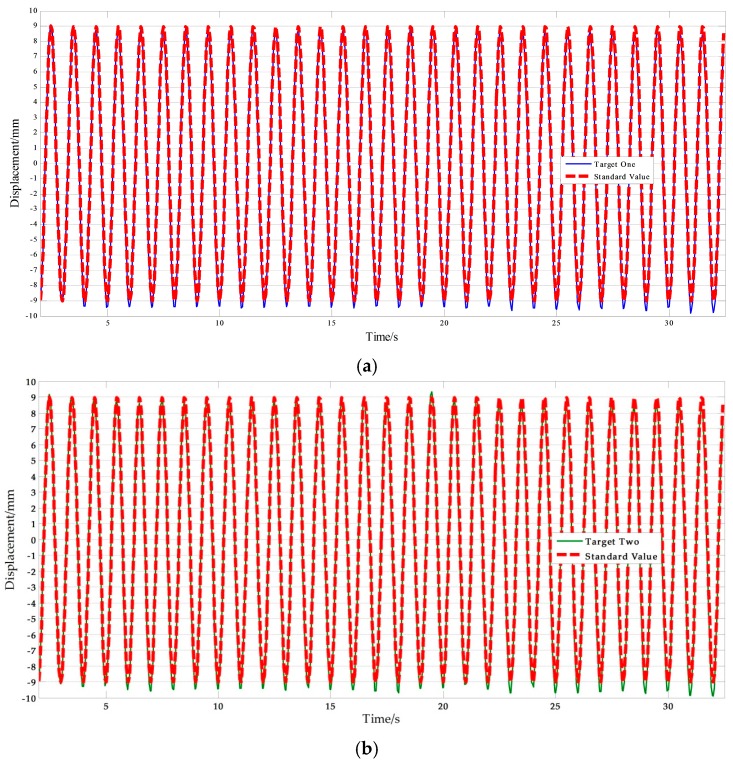
Free target measurement results of vision sensor capturing (**a**) Target one; (**b**) Target two. (*f* = 1.0 Hz, ***A*** = 9 mm)

On the other hand, the average NRMSE error of the target one measurement was 1.753%, and the average NRMSE error of the target two measurement was 1.856%. It can be concluded that the accuracy of the vision sensor measurement is independent of the selected target points. This means that the improved vision-sensor can avoid errors caused by human selection. Furthermore, motion tests with higher frequency were conducted. Figure 14 shows the measurement results and Table 11 lists the NRMSE error analysis results. The maximum NRMSE error of the measurement results of the vision sensor was 3.922%. Measurement accuracy is consistent with the low frequency experimental results. This indicates that the improved vision-based sensor can be applied to track higher frequency motion. It is noteworthy that the performance of the vision sensor in higher frequency measurements depends on the ability of the imaging equipment.

### 4.2. Shaking Table Tests

In order to describe the performance of the vision-based sensor better, a series of higher-frequency and lower-amplitude vibration experiments were carried out to verify the efficiency of this algorithm. A shaking table was used as the vibration source, and the motion was captured by the vision-based sensor and the STDS, just as the moving platform tests mentioned in Section 4.1.

**Figure 15:**
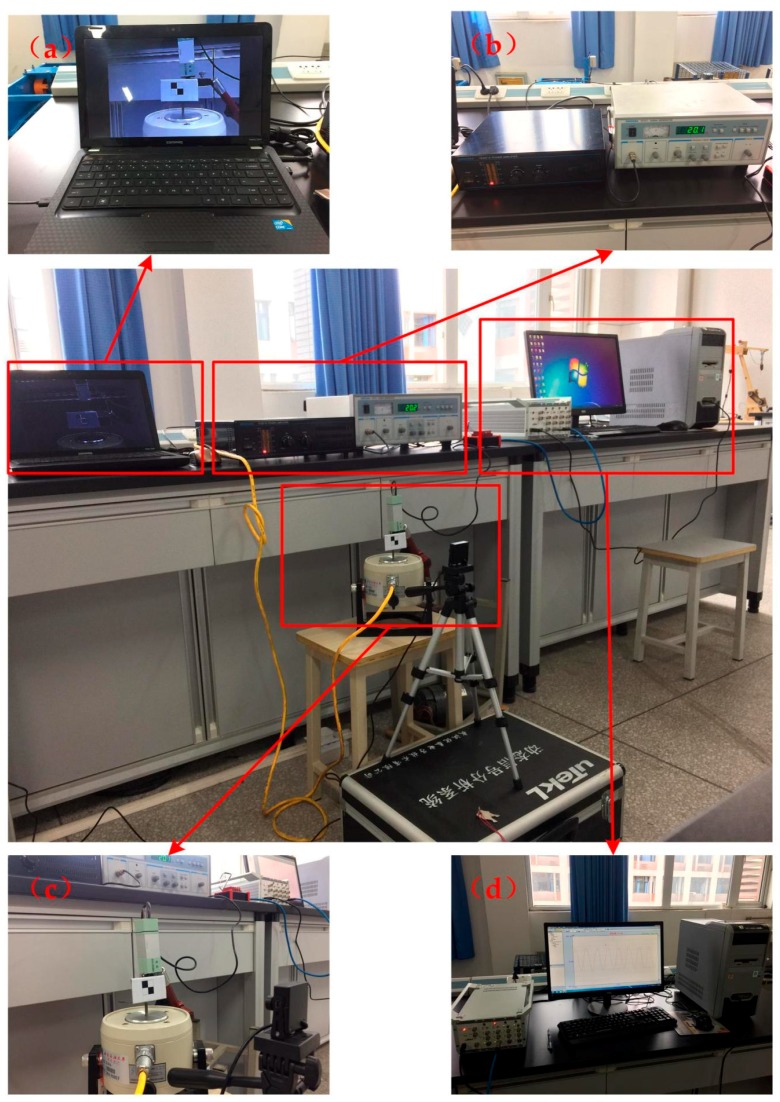
Setup for shaking table experiment: (**a**) Video acquisition system; (**b**) Vibration control system; (**c**) Target system; (**d**) Strain acquisition system.

Figure 15 shows the setup for the shaking table experiment, which includes four components: video acquisition system, vibration control system, target system and strain acquisition system. The video acquisition system are used to capturing the motion states and behaviors, the main role of the vibration control system is controlling the vibration frequency and amplitude while testing, an identifiable target is provided by target system to object tracking steadily and the strain acquisition system is used to collect displacement data obtained by STDS.

**Figure 16:**
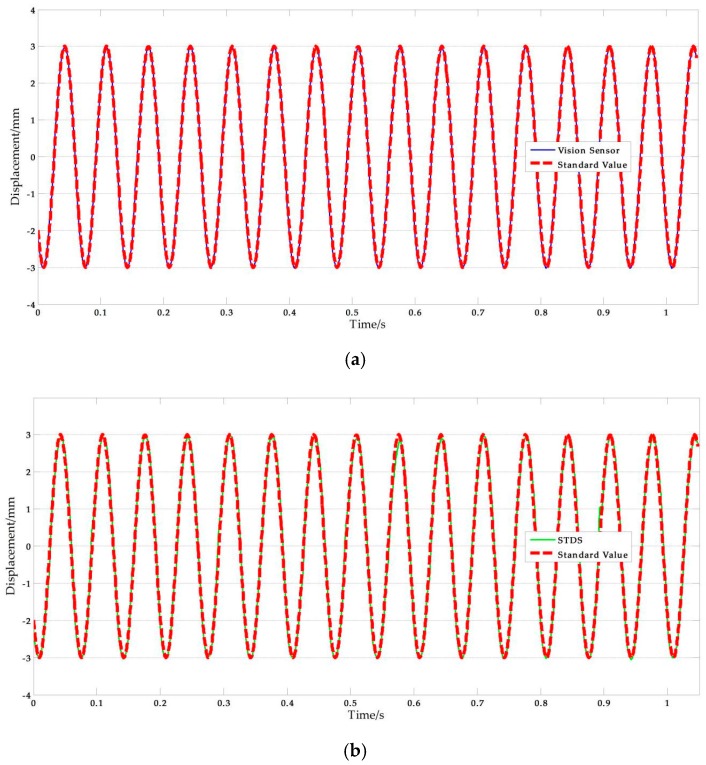
Shaking table test results: (**a**) Measurement results of vision-sensor compared with standard values; (**b**) Measurement results of STDS compared with standard values. (*f* = 15 Hz, *A* = 3 mm).

The experimental parameters of shaking table tests are listed in Table 12, and Figure 16 shows the experimental results of ΙV-9. The NRMSE errors were used in the analysis of experimental data, the results are shown in Table 13. According to the computing results above, we can safely come to the conclusion that: (1) The average value of vision-based sensor measurement error is 2.092%, which is better than STDS, in other words, the performance of vision sensor is better than STDS. (2) The error increases with frequency in the rough while there are some singular values.

### 4.3. Measuring Distance Tests

As a non-contact remote measurement technique, the performance of different measuring distances of the developed vision-based sensors should be analyzed in detail. The different measuring distance are designed to evaluate the impact using shaking table test equipment, the test parameters are listed in Table 14.

**Figure 17:**
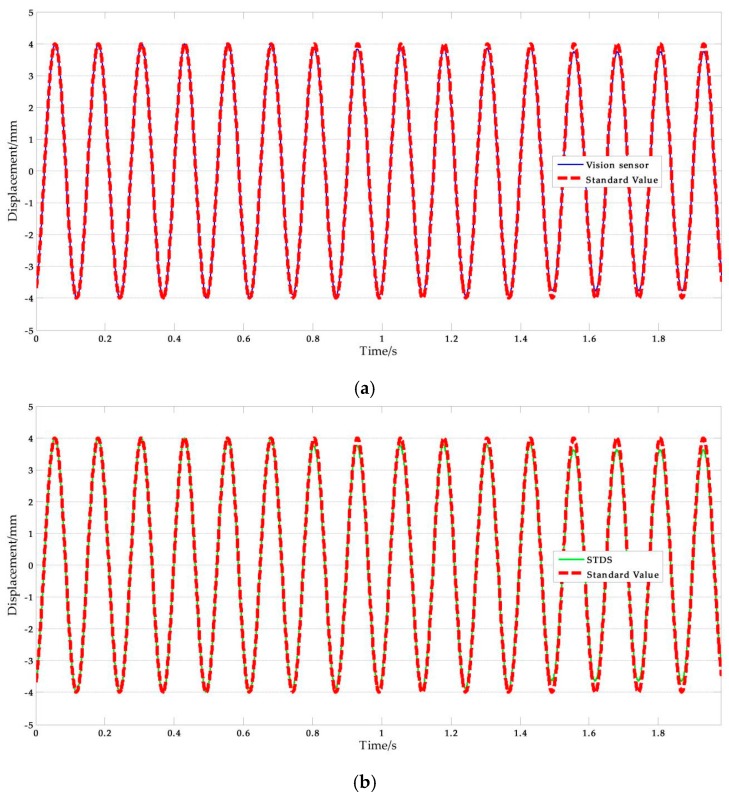
Measuring distance test results: (**a**) Measurement results of vision-sensor compared with standard values; (**b**) Measurement results of STDS compared with standard values. (*D* = 5.0 m).

Figure 17 shows the test results of V-5. The error analysis results are listed in Table 15. The STDS is a kind of connecting displacement sensor and its measuring precision is only affected by frequency and amplitude. On the other hand, the measuring errors of vision sensors increase with the distance. It is well known that the further the distance between target and camera is, the smaller the target is. In other words, the pixel numbers of the target decrease with the distance from the imaging device, providing that the optical focal length is the same. That is the reason which leads to a marked drop in positioning precision, and result in big measuring error. It is worth mentioning that the performance of remote measurement depends on the parameter of imaging equipment, especially the focal length and imaging resolution.

### 4.4. Discussion

From the analysis of the experiments above, it can be seen that the improved object tracking approaches successfully enhance the measurement accuracy of the traditional displacement sensors. Different from the CMT algorithm, the modified CMT algorithm provides more efficient alternatives in vision-based displacement measurements.

First of all, moving platform tests were designed to verify the tracking stability of free targets. Compared with artificial target measurement data acquired in the laboratory, the precision of free target measurement system was verified. The average NRMSE errors from the free target measurement and the artificial target measurement were 1.805% and 1.822%, respectively, which proves that the improved CMT vision measurement algorithm gives a higher accuracy. Two different types of free targets were designed to check whether artificial errors exist in the assignment of initializing region. The NRMSE error between the measuring values target 1# and target 2# was 0.458%, which indicates that no artificial errors appear in this method. From the above two conclusions, we can see that the improved CMT algorithm possesses a good performance on tracking free targets.

Secondly, the moving platform experiments cannot indicate whether this system has a high precision for low amplitude and high frequency vibrations. Therefore, shaking table tests were employed to solve the problem. Compared with mechanical testing and simulation (MTS) electronic servo testsuite, the shaking table can achieve a higher frequency. A series of high frequency and low amplitude vibration tests were designed to further evaluate the performance of the vision sensor. The vibration tests frequency scopes in 8 Hz to 20 Hz and amplitude scopes in 1 mm to 3 mm. Test results prove that the NRMSE error of vision sensor was 2.092%, the results show that the error is within the acceptable level. This demonstrates the reliability of the vision sensors we proposed in high frequency and lower amplitude vibration measurements.

Finally, as a non-contact remote measurement technique, measuring distance was used as a key indicator for judging its performance. Experimental results show that the errors increased with increasing measuring distance and the theoretical analysis indicates that the decisive factor of measuring distance are the characteristics of imaging devices, which are not germane to the algorithm.

## 5. Field Test

**Figure 18:**
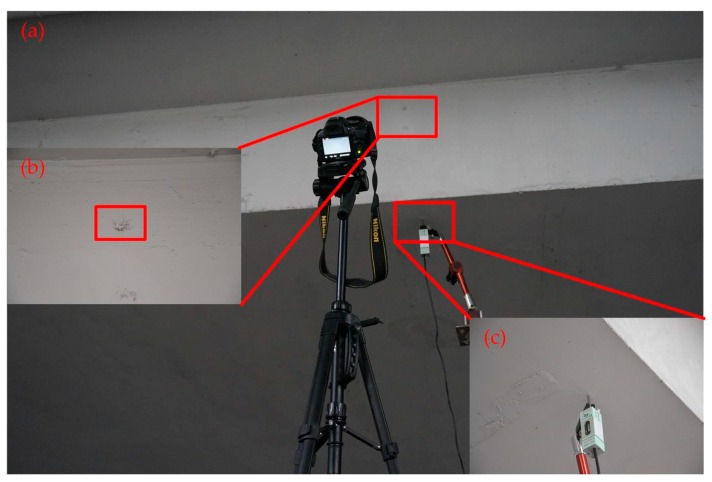
Field test: (**a**) Experiment environment; (**b**) Natural target; (**c**) Displacement by the STDS sensor.

Field tests were carried out to evaluate the validity of the vision sensor on the Yingmenkou flyover of Chengdu (China), which is an important BRT transport hub. The time-domain of motion images was captured by the vision-based sensor and the STDS sensor, respectively. As shown in Figure 18, the vision sensor, limited by the camera optional lens, was installed in a location near the bridge.

Table 16 lists the parameters of the field test and the laboratory test. According to the parameters, the scaling factor was obtained as *SF* = 0.186673.

**Figure 19:**
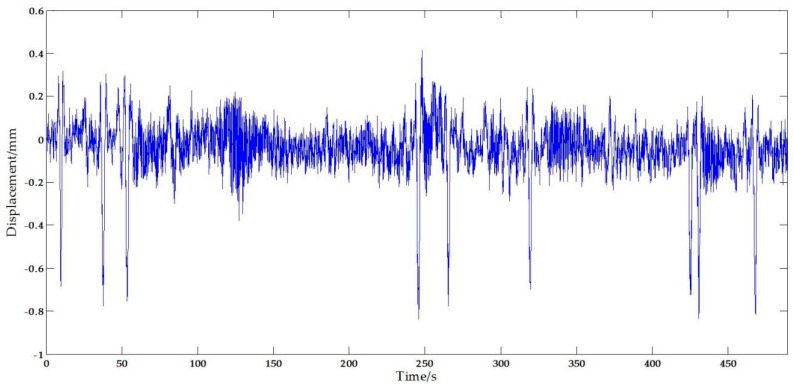
Original vertical displacement from the vision sensor.

Figure 19 plots the displacement measurement from the vision sensor. It can be seen that the measurement results include significant noise signals possibly caused by the movements of the camera stand [46,47], illumination [48] and vapor [48], etc. According to related data and references, the airflow speed has a significant influence on the movements of the stand. The field tests were carried out when the wind speed was lower, so it can be approximately considered that the errors caused by the camera are weak random interfering noise which can be removed by filtering. Similarly, it is proved in the [48] that the illumination and vapor have a great effect on the measurement accuracy of the vision-based system, but scientifically arranging the test to avoid this is not that hard and the trifling impact can be further reduced by a filter. 

**Figure 20:**
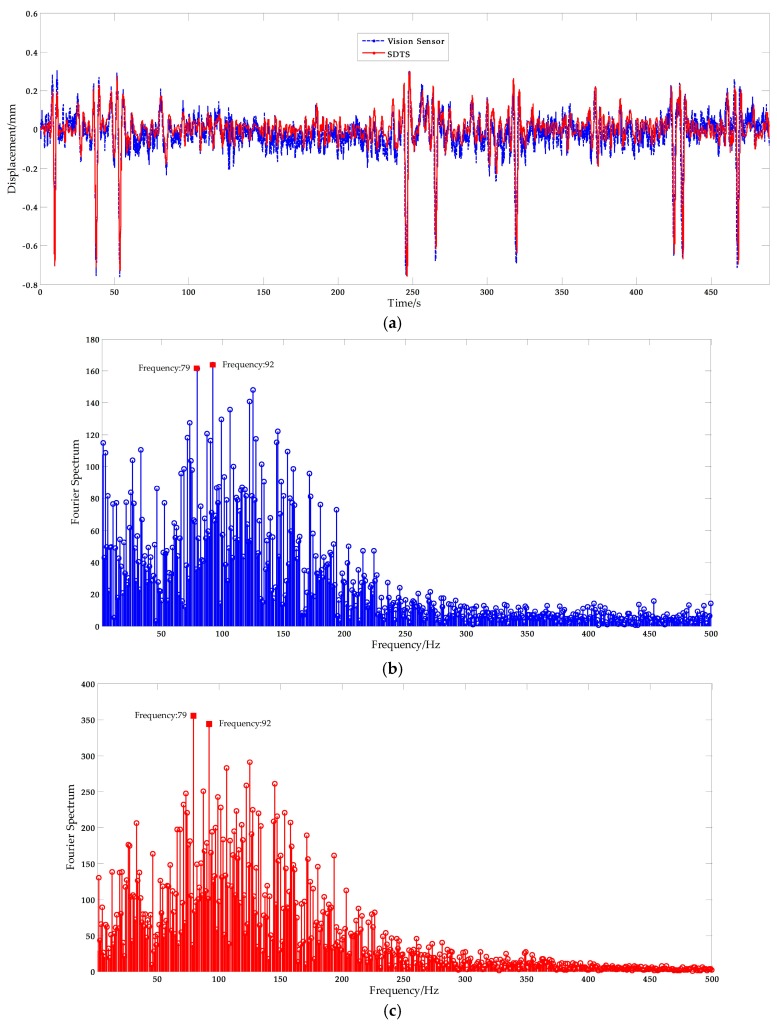
Free excitation of bridge: (**a**) Displacement measurement from the vision sensor and the STDS sensor; (**b**) and (**c**) the corresponding Fourier spectrum results.

In order to obtain the most reliable results, a Butterworth low-pass filter [49] was implemented for noise reduction and the filtering results, plotted in Figure 20a, show that this approach is efficient and useful. Basic displacement characteristics was preserved, while a lot of noise has been filtered. The corresponding Fourier spectrum results are plotted in Figure 20b. The displacement measurement results from the STDS sensor are plotted in Figure 20a, and the Fourier spectrum results are plotted in Figure 20c. The spectral peaks of vision-based sensor measurement results were consistent with the STDS measurement results. The inconsistency of displacement measured by vision sensors and STDS are largely due to the residual noise. From the field test cases, the scaling factor is about 0.14 mm/pixel, and that is, the measurement resolution is ±0.07 mm. Thus the measuring data which between −0.07 mm and 0.07 mm is noisy. That means the residual noise will affect the performance and leads to the difference of curves. Furthermore, two obvious spectral peaks, 79 and 92 Hz, were observed in the Fourier spectrum. Therefore, it can be concluded that the same spectral information can be obtained from the vision sensor.

## 6. Conclusions

In this study, a vision-based sensor system was developed for the BRT viaduct vibration measurement. Combining CMT object tracking algorithm with ORB keypoints detector algorithm, the displacement can be measured with high-precision by tracking any existing target on the structure without the need for pre-installation of an artificial target panel. Detailed experiments, including a series of laboratory tests and a field test, were conducted to evaluate its performance. The following conclusions can be drawn from this study:

- Analysis of different combinations of detectors and descriptors based on the CMT algorithm indicates that the proposed method demonstrated good performance in terms of runtime, CPU usage, and matching accuracy. The realization of the algorithm and the experimental analysis prove that the improved algorithm achieves the same accuracy as comparable methods with less computational cost.
- Based on a detailed analysis of error sources, a synthetical scaling factor calculation method was advanced. The deviation from the tilt angle and lens focal length were reduced, and thus the errors can be well controlled.
- Three laboratory tests were performed to verify the system stability facing free targets and measurement accuracy under the special conditions of low amplitude and high frequency, respectively while exploring the factors influencing distance measuring. Error analysis was performed using the normalized root mean squared error (NRMSE). The possibility of realizing high precision measurements with free targets has been proved. In addition, the maximum spacing between sensing equipment and targets depends on the technical specs and physical parameters, such as optical focal length and resolution.
- The reliability and practicability of the proposed algorithm was validated via actual vibration measurements of a BRT viaduct in Chengdu (China). The precision of the measurement data have been demonstrated by both time and frequency domain data, and thus shows that the proposed vision-based sensors are useful in the on-the-spot working environment.

## Figures and Tables

**Table 1:** Processing time of different combination pairs.

	Detector			
**Descriptor**	**ORB**	**FAST**	**BRISK**	**AGAST**
ORB	3.8349	7.1912	8.4991	7.9967
BRISK	2.7244	7.2786	5.8103	7.6261

**Table 2:** Processor time for different combination pairs.

	Detector			
**Descriptor**	**ORB**	**FAST**	**BRISK**	**AGAST**
ORB	1.2919	4.1020	2.5908	3.9482
BRISK	1.4055	5.2712	2.6885	4.3334

**Table 3:** 1-*Accuracy* of different combination pairs.

	Detector			
**Descriptor**	**ORB**	**FAST**	**BRISK**	**AGAST**
ORB	66.0715	81.8441	49.9039	83.8834
BRISK	52.7826	90.4555	44.5814	89.0591

**Table 4:** Hardware components of proposed vision-based sensor.

Components	Brand	Technical Characteristics	Accessories
Consumer grade camera	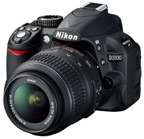 Nikon D3100	Sensor: CMOS	Tripod, lamps, etc.
		Sensor size: 23.1 × 15.4 mm	
		Maximum resolution: 1920 × 1080	
		Image processor: EXPEED 2	
		Interface: HDMI, AV, USB 2.0	
Optical lens	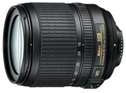 Nikon AF-S DX NIKKOR	Focal length: 18 to 105 mm	
		Maximum aperture: F3.5	
		Minimum focus distance: 0.45 m	
		Manual zoom and luminosity control	
		Dimensions: 89 × 76 mm^2^	
		Weight: 0.42 kg	
Laptop computer	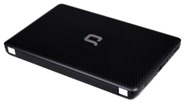 Compaq Presario CQ42	Operating system: Windows 7 × 64	
		CPU: Intel(R) Core(TM) i5 M430 @ 2.27 GHz	
		RAM: 4 GB DDR3	
		Discs: Seagate 500 GB 5400 rpm 16 MB	
		Screen: 14″	

**Table 5:** Experimental parameters of artificial target tests.

Number	Frequency (Hz)	Amplitude (mm)	Operating Mode
Ι-1	0.05	±5	Sine-wave
Ι-2	0.10	±5	Sine-wave
Ι-3	0.05	±5	Liner-wave
Ι-4	0.10	±5	Liner-wave
Ι-5	0.05	±9	Sine-wave
Ι-6	0.10	±9	Sine-wave
Ι-7	0.05	±9	Liner-wave
Ι-8	0.10	±9	Liner-wave

**Table 6:** Experimental parameters of free target tests.

Number	Frequency (Hz)	Amplitude (mm)	Operating Mode
II-1	0.05	±5	Sine-wave
II-2	0.10	±5	Sine-wave
II-3	0.05	±5	Liner-wave
II-4	0.10	±5	Liner-wave
II-5	0.05	±9	Sine-wave
II-6	0.10	±9	Sine-wave
II-7	0.05	±9	Liner-wave
II-8	0.10	±9	Liner-wave

**Table 7:** Experimental parameters of higher frequency vibration tests.

Number	Target Type	Frequency (Hz)	Amplitude (mm)	Operating Mode
III-1	Artificial	0.5	±9	Sine-wave
III-2	Artificial	1.0	±9	Sine-wave
III-3	Natural	0.5	±9	Liner-wave
III-4	Natural	1.0	±9	Liner-wave

**Table 8:** Laboratory test cases.

*x_A_-x_B_* (mm)	$I_{A}^{i}$*-*$I_{B}^{i}$ (Pixel)	Measurement Distance (mm)	Focal Length (mm)	*d_pixel_* (mm/Pixel)
11.5	80	528.2	55.15	0.0144

**Table 9:** NRMSE analysis of artificial target experimental results.

Number	NRMSE between Vision Sensor Measured Values and Standard Values (*%*)	NRMSE between STDS Measured Values and Standard Values (*%*)
Ι-1	1.630	0.945
Ι-2	3.041	3.433
Ι-3	1.125	0.919
Ι-4	2.757	2.068
Ι-5	0.768	0.726
Ι-6	1.549	0.850
Ι-7	1.547	0.951
Ι-8	1.272	1.646
Avgerage	1.822	1.442

**Table 10:** NRMSE analysis of free target experimental results.

Number	NRMSE between Target One Measured Values and Standard Values (*%*)	NRMSE between Target Two Measured Values and Standard Values (*%*)	NRMSE between STDS Measured Values and Standard Values (*%*)	NRMSE between Target One Measured Values and Target Two Measured Values (*%*)
II-1	3.231	3.218	0.731	1.005
II-2	2.184	2.375	0.796	0.472
II-3	1.778	1.765	1.568	0.331
II-4	2.075	2.276	3.404	0.705
II-5	0.836	1.301	0.619	0.353
II-6	1.983	2.120	1.112	0.268
II-7	0.800	0.787	1.470	0.284
II-8	1.140	1.005	2.069	0.243
Avgerage	1.753	1.856	1.471	0.458

**Table 11:** NRMSE analysis of free target experimental results with higher frequency.

Target Point Type	Frequency (Hz)	NRMSE between Measured Values and Standard Values (*%*)	
**Artificial target**	0.5	0.651	
	1.0	0.569	
**Free targets**	0.5	0.724	0.765
	1.0	0.523	0.762

**Table 12:** Experimental parameters of shaking table tests.

Number	Frequency (Hz)	Amplitude (mm)	Operating Mode
IV-1	8	±1	Sine-wave
ΙV-2	8	±2	Sine-wave
ΙV-3	8	±3	Sine-wave
ΙV-4	10	±1	Sine-wave
ΙV-5	10	±2	Sine-wave
ΙV-6	10	±3	Sine-wave
ΙV-7	15	±1	Sine-wave
ΙV-8	15	±2	Sine-wave
ΙV-9	15	±3	Sine-wave
ΙV-10	20	±1	Sine-wave
ΙV-11	20	±2	Sine-wave
ΙV-12	20	±3	Sine-wave

**Table 13:** NEMSE errors analysis of shaking table experimental results.

Number	Frequency (Hz)	Amplitude (mm)	NRMSE between Vision Sensor Measured Values and Standard Values (*%*)	NRMSE between STDS Measured Values and Standard Values (*%*)
IV-1	8	±1	0.842	1.657
ΙV-2	8	±2	0.699	1.556
ΙV-3	8	±3	1.445	2.965
ΙV-4	10	±1	1.668	2.975
ΙV-5	10	±2	2.391	3.889
ΙV-6	10	±3	1.876	2.096
ΙV-7	15	±1	2.899	3.764
ΙV-8	15	±2	2.567	4.041
ΙV-9	15	±3	2.912	2.975
ΙV-10	20	±1	2.081	2.999
ΙV-11	20	±2	2.678	5.014
ΙV-12	20	±3	3.043	4.064
Average			2.092	3.166

**Table 14:** Experimental parameters of measuring distance tests.

Number	Frequency (Hz)	Amplitude (mm)	Measuring Distance (m)	Operating Mode
V-1	8	±4	1.0	Sine-wave
V-2	8	±4	2.0	Sine-wave
V-3	8	±4	3.0	Sine-wave
V-4	8	±4	4.0	Sine-wave
V-5	8	±4	5.0	Sine-wave
V-6	8	±4	6.0	Sine-wave
V-7	8	±4	7.0	Sine-wave
V-8	8	±4	8.0	Sine-wave
V-9	8	±4	9.0	Sine-wave
V-10	8	±4	10.0	Sine-wave
V-11	8	±4	11.0	Sine-wave
V-12	8	±4	12.0	Sine-wave

**Table 15:** NEMSE errors analysis of measuring distance experimental results.

Number	Measuring Distance (m)	NRMSE between Vision Sensor Measured Values and Standard Values (*%*)	NRMSE between STDS Measured Values and Standard Values (*%*)
V-1	1.0	1.035	1.632
V-2	2.0	0.889	0.894
V-3	3.0	0.924	2.119
V-4	4.0	1.368	3.186
V-5	5.0	1.789	1.873
V-6	6.0	2.583	2.575
V-7	7.0	3.786	1.645
V-8	8.0	3.357	1.563
V-9	9.0	3.527	2.087
V-10	10.0	5.068	1.563
V-11	11.0	6.268	1.877
V-12	12.0	7.329	1.771

**Table 16:** Field test cases.

*x_A_-x_B_* (mm)	$I_{A}^{i}$-$I_{B}^{i}$ (Pixel)	Measurement Distance (mm)	Focal Length (mm)	Tilt Angle (°)	*d_pixel_* (mm/Pixel)
13.84	72	469	42.95	22	0.01426
